# International Breastfeeding Journal: Introducing a new journal

**DOI:** 10.1186/1746-4358-1-1

**Published:** 2006-03-09

**Authors:** Lisa H Amir

**Affiliations:** 1 Key Centre for Women's Health in Society, University of Melbourne, Australia

## Abstract

*International Breastfeeding Journal *is a new open access peer-reviewed journal with a multidisciplinary focus. The aim of *International Breastfeeding Journal *is to contribute to understanding all aspects of breastfeeding. Breastfeeding is recognized as an important public health issue with enormous social and economic implications. In order to help women breastfeed successfully there is a need to understand both the physiology of lactation and the social and cultural context within which breastfeeding occurs. *International Breastfeeding Journal *invites manuscripts from around the world, which address all of these aspects, including the impediments to breastfeeding, the health effects of not breastfeeding for infants and their mothers, and the management of breastfeeding problems.

## Editorial

Welcome to *International Breastfeeding Journal*, a new open access peer-reviewed journal with a multidisciplinary focus.

My interest in breastfeeding was sparked when I became a mother in 1984 and began to learn about feeding infants. I was intrigued by the pioneering studies of Niles Newton in which she experimented on the effects of stress on the let-down reflex – on herself [1]. I was unsettled by Maureen Minchin's book *Breastfeeding Matters: What We Need to Know About Infant Feeding*, first published in 1985, in which she described her own experiences of struggling through breastfeeding problems in 1976 [2] until she came across Mavis Gunther's earlier work on nipple pain [3,4].

As a medical student in the 1970s I had received one lecture on infant feeding – with an emphasis on artificial feeding. Breastfeeding rates were low in many developed countries in the mid 1970s, until an interest in natural childbirth and infant feeding resurfaced and mother-to-mother support groups, such as the La Leche League International (LLLI) and the Nursing Mothers' Association of Australia (now the Australian Breastfeeding Association) began grass-roots movements to help mothers breastfeed. By the mid-eighties, volunteer breastfeeding counsellors, such as Chele Marmet realized that it was time to create a new occupation: lactation consulting [5]. The International Board of Lactation Consultant Examiners administered the first examination in 1985, and by the time of the 2005 examination there were nearly 15,000 currently certified International Board Certified Lactation Consultants (IBCLCs) worldwide, in 65 countries [6]. JoAnne Scott worked tirelessly as IBLCE's executive director from its inception until early 2005 [7]. The professional organisation for IBCLCs, the International Lactation Consultants Association, has recently celebrated 20 years as a new profession with a conference for over 900 participants in Chicago, IL, USA [8] – where LLLI had begun in 1956.

The medical profession had shown little interest in breastfeeding, but pioneers such as Derrick B Jelliffe published *Human Milk in the Modern World: Psychosocial, Nutritional and Economic Significance *[9] in 1978 and Ruth Lawrence published the first edition of *Breastfeeding: A Guide for the Medical Profession *in 1979 [10]. In Africa, Felicity Savage worked to improve health workers' knowledge of breastfeeding [11,12]. In another ground-breaking step, Jack Newman set up one of the first hospital-based breastfeeding clinics in Canada in 1984 at Toronto's Hospital for Sick Children. An organisation for physicians dedicated to the protection, promotion and support of breastfeeding was founded in 1994. The Academy of Breastfeeding Medicine holds an annual meeting and has developed a range of protocols aimed at managing common medical problems that may impact breastfeeding success [13].

Where would we be without the researchers who began to study breastfeeding and reassess what we "know"? A few of these immediately come to mind: the physiologists such as Roger Short [14], Michael Woolridge [15,16] and Peter Hartmann and colleagues [17] and immunologists such as Lars Hanson [18]; the midwives, Chloe Fisher [19], Mary Renfrew [20] and Sue Cox [21]; the nurses, Paula Meier [22] and Gene Cranston Anderson [23]; the social scientists such as Kathy Dettwyler [24]); and economists, such as Marilyn Waring who taught us the worth of women's milk [25]. More recently, epidemiologists have shown us how to conduct studies into the long-term effects of infant feeding: Michael Kramer and colleagues have conducted a cluster randomised controlled trial, where the unit of randomisation was the maternity hospital [26]. We also need to acknowledge the work done by the World Health Organization and UNICEF, in particular to improve maternity care via the Baby-Friendly Hospital Initiative [27].

The multidisciplinary nature of breastfeeding can be seen by looking at the professions that have been represented in this short list. However, many important professions have been left out because of the brief nature of my review: dieticians, speech pathologists, community nurses, general practitioners, psychologists, dentists, policy makers and others.

*International Breastfeeding Journal *is an online journal, which aims to facilitate communication between researchers, clinicians and activists working in all of these fields. Apart from some cultural overlay, lactation, breasts, babies and breastfeeding are all pretty much the same worldwide. The IBCLCs, the mother-to-mother support counsellors, the health professionals and all the others who support breastfeeding in their own way – all deserve open access to quality information on breastfeeding.

The journal is based on the factual scientific premise that breastfeeding is the normal or "default" method of infant feeding [28]. Thus breastfeeding is not beneficial to the health of infants or mothers, it is artificial feeding which is a harmful practice [29]. We acknowledge that not all mothers will be able to provide human milk for their infants, so we need to study how to best feed these infants. Human milk banking can be a safe second-best if mother's own milk is not available [30]. Milk banks need to become as common as blood banks. Artificial feeds continue to be a risk to infant health [31], and further efforts are needed to improve the quality of production, even basic microbiological safety [32], of these critical food sources. Research on the inter-generational effects of human infant feeding is overdue.

*International Breastfeeding Journal *is a peer-reviewed journal, supported by a strong Editorial Board. All papers are reviewed by at least two experts in the field. Articles published in *International Breastfeeding Journal *will be listed in PubMed and permanently archived in PubMed Central as well as certain other national archives.

## Journal scope

*International Breastfeeding Journal *contributes to understanding all aspects of breastfeeding. Breastfeeding is recognized as an important public health issue with enormous social and economic implications. In order to help women breastfeed successfully there is a need to understand both the physiology of lactation and the social and cultural context within which breastfeeding occurs. *International Breastfeeding Journal *seeks to address all of these aspects, including the impediments to breastfeeding, the health effects of not breastfeeding for infants and their mothers, and the management of breastfeeding problems.

The journal will consider the following article types: research, reviews, case reports, study protocols, short reports, methodology, commentaries, hypotheses, and debate articles.

## Open access

*International Breastfeeding Journal *is an open access journal, which means:

• All articles will be freely and universally accessible online without any barriers to access, which increases their visibility.

• You and your peers will be free to print out copies of your article, email it to colleagues, and post it on the web because of the BioMed Central copyright and license agreement.

Open access journals are funded by article processing charges (APCs) rather than journal subscriptions. The costs are, therefore, borne by the authors, their institutions or their research grants, and access to the journals is free of charge via the web.

BioMed Central membership offers a way for institutions to support open access publishing and reduce the cost of the APC borne by the author. This scheme enables BioMed Central member institutions to pay a fee that ensures their researchers are wholly or partially exempt from APCs. BioMed Central membership has a number of benefits. In addition to the direct benefits in terms of discounted or waived APCs there are public policy benefits in supporting an open access journal regime such as BioMed Central. Open access journals are one mechanism for putting pressure on regular journal publishers to moderate their price increases.

There are several types of membership available tailored to the varying needs of BioMed Central's customers [33].

Please note that no article processing charges will be payable on manuscripts submitted in the first six months following the launch of the journal. Article processing charges are also usually regarded as a legitimate charge against research grants. In the medium term, alternative arrangements, such as institutional support, should be encouraged, although after this time the Editor-in-Chief will be able to grant a limited number of discretionary processing charge waivers.

## Competing interests

LHA is a member of the Academy of Breastfeeding Medicine, Australian Breastfeeding Association, Australian Lactation Consultants Association, International Lactation Consultants Association, Public Health Association of Australia and the Royal Australian College of General Practitioners.
